# Knowledge, attitudes, and practices toward COVID-19: A cross-sectional study during normal management of the epidemic in China

**DOI:** 10.3389/fpubh.2022.913478

**Published:** 2022-09-08

**Authors:** Juan Yang, Yuting Liao, Qianhui Hua, Chang Sun, Huakun Lv

**Affiliations:** ^1^State Key Laboratory of Molecular Vaccinology and Molecular Diagnostics, National Institute of Diagnostics and Vaccine Development in Infectious Diseases, School of Public Health, Xiamen University, Xiamen, China; ^2^School of Medicine, Ningbo University, Ningbo, China; ^3^School of Journalism and Communication, Peking University, Beijing, China; ^4^The Center for Disease Control and Prevention of Zhejiang Province, Hangzhou, China

**Keywords:** COVID-19 vaccine, online survey, knowledge, attitude, practices

## Abstract

**Background:**

The COVID-19 pandemic is striking the world with serious public health and economic losses. Complying with precautionary measures is affected by knowledge, attitudes, and practices (KAP) toward COVID-19 among the general public, so it is urgent to know the public's awareness of COVID-19 as to promote the epidemic management of COVID-19 in China.

**Methods:**

An online sample of Chinese residents was recruited. We administered a self-developed online KAP survey comprising 39 questions regarding awareness of COVID-19, transmission mode, symptoms, preventive measures, and respondents' attitudes and practices with respect to COVID-19. The total score of each item (knowledge, attitudes, and practices) adopts the ten points system, score of KAP is 30 points. Descriptive statistics, analysis of variance, and binomial logistic regression were used in the statistical analysis.

**Results:**

Among respondents, average scores for COVID-19-related knowledge, attitudes, and practice were 8.94 ± 0.79, 5.97 ± 1.58, and 7.03 ± 3.14, respectively. 91.2% were aware that COVID-19 is an acute viral infection and 99.95% knew that wearing a mask is one way to prevent COVID-19 infection. Participants correctly identified the symptoms of COVID-19 with a high accuracy rate of over 85%.

**Conclusion:**

Many adults in the present study had adequate knowledge, a positive attitude and engaged in correct practices against COVID-19. People in China have a high awareness of epidemic prevention and control. However, conducting KAP surveys among people with different demographic characteristics at different stages of the epidemic is important to improve public health education and implement proper COVID-19 prevention and control measures.

## Introduction

Severe acute respiratory syndrome coronavirus 2 (SARS-CoV-2) is the causative agent of the respiratory illness known as coronavirus disease 2019 (COVID-19). COVID-19 could lead to serious respiratory conditions, the main clinical signs and symptoms include fatigue, high fever, dry cough, dyspnea, fatigue and myalgia. It could lead to severe pneumonia, acute respiratory syndrome, and even death in some severe cases (1, 2). Since emerging in December 2019, the outbreak of COVID-19 has spread to nearly every country worldwide (3). On 11 March 2020, the World Health Organization declared the COVID-19 epidemic to be a pandemic (4). As of 4 April 2022, there have been more than 489 million cumulative COVID-19 cases and 6 million deaths worldwide (5). The COVID-19 pandemic in China is largely under control, but local outbreaks of COVID-19 have occurred in parts of the country including Beijing, Wuhan, and Hebei (6–8), indicating that disease prevention and control measures must continue to be followed in the country. The public is one of the key actors in Public Health Emergency Preparedness, responding to Public Health Emergency by heightened risk perceptions (9, 10), increased knowledge and awareness about specific threats (11), and the implementation of precautionary measures (12, 13). Morever, recent studies on COVID-19 revealed knowledge perceived controllability, optimistic beliefs, emotion, and risk perception might all interpret precautionary actions of the public (14–17).

In addition, the knowledge, attitudes, practices (KAP) model is commonly used to explain how individual knowledge and attitudes affect health behavior changes, which is a behavioral intervention theory. It was proposed in the 1960s by Mayo, a professor at Harvard University (18). The improvement of personal knowledge, attitudes, and practices can help improve health-related behavior as well as disease prevention and control (19). Therefore, understanding people's knowledge, attitudes, and behaviors toward COVID-19 can provide a reference for the development of health education plans. One study revealed that adherence to prevention and control measures is an essential strategy to halt the spread of an infectious disease outbreak (14), which is directly linked to the knowledge, attitudes, and practices (KAP) level of the population toward COVID-19. According to Khattak et al., sex, marital status, education, and residential area have a significant association with COVID-19-related knowledge scores (20). A study among adolescents in Spain showed that COVID-19-related knowledge was influenced by sex, place of residence, level of education, and financial aid; attitudes and risk perceptions were influenced by age and financial aid (21). A study conducted by Kebede et al. showed that COVID-19 risk communication and public education efforts should focus on building an appropriate level of knowledge while enhancing the adoption of recommended self-care practices, with special emphasis on high-risk audiences segments (22). A study in Sierra Leone shows that in the context of COVID-19, there is a strong association between knowledge and practices (23). People's knowledge, attitude and practices (KAP) toward COVID-19 may play a critical role in their acceptance of measures to curb its spread and their willingness to seek and adhere to treatment (24). Research in Uganda has shown that there are differences in perceptions of COVID-19 across occupations and the need to mobilize the entire population to the same level of knowledge will have an impact on attitudes and practices to prevent the spread of COVID-19 disease (25).

Our research was carried out at a time when COVID-19 had been brought under control in China and regular work and production had returned to normal levels, but there was still a risk of sporadic and imported cases. With recovery of the economy, population mobility increases and the task of epidemic prevention and control becomes challenging. Thus, the aim of this study was to evaluate the KAP regarding COVID-19 among residents of China and factors affecting KAP and provide a basis for relevant authorities to formulate effective prevention and control strategies, so as to promote correct information regarding COVID-19 control among the general public and improve their capacity toward COVID-19 prevention, to facilitate COVID-19 outbreak management.

## Materials and methods

### Study design, participants, and sampling

We conducted a cross-sectional survey using convenience and snowball sampling from September to October 2020 in Zhejiang Province, participants aged over 18 could fill in the questionnaire anonymously. The survey conducted through the largest online survey platform in China, Wen Juan Xing (https://www.wjx.cn/ accessed 4 April 2022). We consulted the relevant domestic and foreign literature on vaccination willingness and referred to relevant questionnaires with high reliability and validity; together with information related to COVID-19 infection, COVID-19 vaccination, and China's social and cultural background, we designed the questionnaire used in this investigation. The online survey link was disseminated *via* QQ (https://im.qq.com/index accessed 4 April 2022) and WeChat (https://weixin.qq.com/ accessed 4 April 2022), on which personal information and public websites can be shared with family members, friends and colleagues and forwarded to others by participants. To identify possible problems with our self-developed questionnaire, we conducted a preliminary survey among a small group of people before formally administering the survey. The results of returned survey and deficiencies described in feedback regarding survey items were revised, and the questionnaire was improved on this basis. We used a formula $\text{n}={\left(\frac{{\text{Z}}_{\left(1-\frac{\alpha }{2}\right)}}{\text{d}}\right)}^{2}\text{p}\left(1-\text{p}\right)\times \text{Deff}$ of sample size required for cross-sectional investigation, the willingness rate of COVID-19 vaccination is 80%, significance level α=0.05, and absolute allowable error *d* = 2.5%. As the research was non-random sampling, Deff =2 was taken as the design effect. Considering the sample loss caused by unpredictable factors, we increased by about 10% on the basis of the estimated sample size, and the minimum was calculated as *n* = 2164. To ensure validity of the online survey, we applied the following exclusion criteria: incomplete answers, inconsistent answers, and obvious logic errors responses. At last, 12 invalid questionnaires that did not meet the requirements were excluded, leaving a total of 2,171 valid questionnaires.

### Data collection instrument

The questionnaire included basic demographic characteristics and information regarding knowledge, attitude, and practice levels. The knowledge section comprised four parts: awareness about COVID-19, a total of 10 items; transmission mode of COVID-19, seven items in total; symptoms of COVID-19 infection, 8 items in total; and preventive measures against COVID-19 infection, nine entries in total. Respondents rated the statements as “true,” “false” and “unclear.” The total score in each of the four dimensions was 10 points. The total score for the knowledge section was also 10 points, which was the average of the total scores in the four dimensions. The attitude section comprised four questions: “Do you think the domestic COVID-19 epidemic will worsen again in autumn and winter this year?” “Do you think you will be infected with COVID-19 this autumn and winter?”, “What is your opinion regarding the safety of the COVID-19 vaccines currently entering phase III clinical trials in China?”, “What is your opinion regarding the effect of the COVID-19 vaccines that has entered phase III clinical trials in China?” The practice section included one question: “Would you be vaccinated once the COVID-19 vaccines receive an Emergency Use Administration authorization?” For questions related to knowledge, correct responses were scored as one point; incorrect and unclear responses were scored as zero point. In the attitude and practice segments, each question was assigned a score from 0 to 10, with zero being the least likely/strongly disagree and 10 being the most likely/strongly agree.

### Statistical analysis

Microsoft Excel 2016 (Microsoft Corporation, Redmond, WA, USA) was used to sort the data, and IBM SPSS 24.0 (IBM Corp., Armonk, NY, USA) was used for statistical analysis. Classification variables are expressed as frequency and percentage, and continuous variables are expressed as mean and standard deviation. Single factor analysis was performed using *F* or *t-tests*. The variables age, sex, education level, average monthly income, and occupation were included in the multivariate binary or ordered multiple logistic regression analysis model (forward logistic regression). A two-sided test was performed with test level α = 0.05. “Wald test” was used to test the regression coefficient of the Logistic regression model, we conducted “Hosmer and lemeshow Tests” on logistic regression models. The four models (knowledge, attitude, practice, KAP) worked well (*p* > 0.1).

## Results

In total, 2171 valid questionnaires were finally included in the analysis, our participants came from all prefecture-level cities in the province, covering a wide range. Most respondents were women (60.5%). Respondents aged 18–30, 31–40, 41–50, and ≥51 years accounted for 29.4, 32.2, 24.3, and 14.1% of the sample, respectively. Among respondents, 71.8% had a junior college/university degree, 11.7% had a technical secondary school degree or below, and 16.5% had a master's degree or above. Among respondents, 38.7% were students; those engaged in mental labor, other occupations, and physical labor accounted for 23.8, 24.1 and 13.4% of the sample, respectively. Respondents with monthly income between 5001 and 10,000 RMB accounted for the largest proportion (38.7%).

In the knowledge section of the survey, 42.6% of respondents correctly answered all 10 items in the first part, awareness about COVID-19. The correct response rate of items “COVID-19 is an acute viral infection,” “COVID-19 is spreading worldwide and can infect anyone in any age,” “COVID-19 is a serious disease that can cause death,” “COVID-19 infection is more serious and has a higher mortality rate in older people,” and “Contracting COVID-19 is more severe for people with chronic diseases, with higher mortality rates” were above 90%. The correct response rate for “COVID-19 cannot spread among people easily,” “COVID-19 is not a serious public health problem,” and “There are specific drugs for the treatment of COVID-19” were very low, with 1.38, 3.59, and 7.55%, respectively. The correct response rate for “COVID-19 affects the economy by reducing labor productivity and increasing the burden of healthcare costs,” and “Vaccination is the most effective way to prevent COVID-19 and its complications” were 81.85% (Table 1).

**Table 1:** Cognition of COVID-19.

**Items**	**Accuracy (%)**
K1: COVID-19 is an acute viral infection	91.20
K2: COVID-19 is spreading worldwide and can infect anyone in any age	99.12
K3: COVID-19 cannot spread among people easily	1.38
K4: COVID-19 is a serious disease that can cause death	94.10
K5: COVID-19 is not a serious public health problem	3.59
K6: COVID-19 affects the economy by reducing labor productivity and increasing the burden of healthcare costs	81.85
K7: COVID-19 infection is more serious and has a higher mortality rate in older people	92.95
K8: Contracting COVID-19 is more severe for people with chronic diseases, with higher mortality rates	92.03
K9: There are specific drugs for the treatment of COVID-19	7.55
K10: Vaccination is the most effective way to prevent COVID-19 and its complications	81.85

In the second part, mode of COVID-19 transmission, only 330 respondents (15.20%) correctly responded to all seven items. “transmission by droplets from an infected person” item had the highest correct response rate (99.59%), and “contacting pets” item had the lowest rate (51.08%). The correct response rates for “touching elevators, tables, door handles, handrails, coins or paper money,” “exposure to fecal contaminants,” “frozen food imported from abroad,” and “aerosols” were more than 85%, and that of “shaking hands with an infected person” was slightly lower (79.92%) (Table 2).

**Table 2:** Transmission mode of COVID-19.

**Propagation mode of COVID-19**	**Accuracy (%)**
K11: Transmission by droplets from an infected person (talking, coughing, sneezing)	99.59
K12: Shaking hands with an Infected person	79.92
K13: Touch elevators, tables, door handles, handrails, coins or paper money	88.95
K14: Contact pets	51.08
K15: Exposure to faecal contaminants (public toilets)	87.47
K16: Frozen food imported from abroad (seafood, meat)	86.27
K17: Aerosols (aerosols formed when droplets are mixed in the air and can cause infection when inhaled)	95.49

In the third part of the knowledge section, symptoms and manifestations of COVID-19 infection, 77.34% of respondents correctly responded to all eight items. The correct response rate for “fever,” “cough,” “fatigue,” “pharyngalgia,” “myalgia,” and “dyspnea” were over 90%. “rhinobyon” item had the lowest rate of correct responses (85.17%) (Table 3).

**Table 3:** Symptoms and manifestations of COVID-19 infection.

**COVID-19 infection symptoms or manifestations**	**Accuracy (%)**
K18: Fever	98.85
K19: Cough	97.60
K20: Fatigue	98.11
K21: Rhinobyon/running nose	85.17
K22: Pharyngalgia	92.49
K23: Myalgia	92.40
K24: Vomiting/diarrhea	87.98
K25: Dyspnea	97.47

In the last part of this section, preventive measures against SARS-CoV-2 infection, 55.69% of respondents answered all nine questions correctly. The correct response rate of “taking antibiotics,” and “eating garlic” were only 14.37 and 17.55%, respectively. Some respondents had the misperception that eating garlic and taking antibiotics could prevent COVID-19. The correct response rate for the remaining seven items exceeded 95% (Table 4).

**Table 4:** Preventive measures for SARS-CoV-2 infection.

**Preventive measures for SARS-CoV-2 infection**	**Accuracy (%)**
K26: Frequent hand-washing	99.77
K27: Keep a distance from people	99.72
K28: Avoid rubbing eyes, mouth and nose	98.89
K29: Wear a mask in public	99.95
K30: Try not to touch elevator buttons, door handles and other public facilities directly	99.12
K31: Ventilate the room	99.36
K32: Take antibiotics	14.37
K33: Eat garlic	17.55
K34: COVID-19 vaccination	95.12

### Univariate analysis of COVID-19-related KAP scores

For COVID-19 knowledge scores, the average score was 8.94 ± 0.79. The mean ± standard deviation for COVID-19 knowledge scores was 8.88 ± 0.87 in men and 8.97 ± 0.74 in women (*P* = 0.014). Knowledge scores among respondents aged 31–50 years were higher than those among respondents between age 18–30 years and those over 50 years of age (*P* < 0.001). Respondents with higher education levels had higher COVID-19 knowledge scores, with the highest mean score among those with a master's degree or above (9.03 ± 0.76, *P* < 0.001). Participants with higher monthly income also had higher scores, with the highest mean score among those with monthly income more than 10,000 RMB (8.98 ± 0.78, *P* = 0.007). Mental laborers had the highest mean score (9.06 ± 0.74) for COVID-19 knowledge; those with other occupations and physical workers had lower scores (8.78 ± 0.82 and 8.81 ± 0.87, respectively; *P* < 0.001). Thus, sex, age, education, monthly income, and occupation were factors significantly associated with COVID-19 knowledge score.

For attitudes regarding COVID-19, the average score was 5.97 ± 1.58; this score was higher among men (6.16 ± 1.61) than among women (*P* < 0.001). Older respondents had higher attitude scores, with the highest scores among respondents aged more than 50 years (*P* < 0.001). Respondents with a technical secondary school education and below had the highest attitude scores. Interestingly, the higher the level of education, the lower the COVID-19 attitude score (*P* < 0.001). Physical laborers had the highest mean attitude score (6.08 ± 1.51); mean scores among mental laborers and students were 6.00 ± 1.58 and 5.60 ± 1.31, respectively (P=0.008). Sex, age, education level, and occupation were factors significantly associated with COVID-19 attitude scores.

Regarding practices related to COVID-19, the average score was 7.03 ± 3.14, and mean scores were higher among men (7.50 ± 3.01) than women (*P* < 0.001), Respondents aged between 41 and 50 years had the highest practice scores whereas those aged 18–30 years had the lowest scores. Scores were similar among respondents over 50 years old and those aged 41–50 years (*P* < 0.001). Mean practice scores among respondents living in urban and rural areas were 6.91 ± 3.18 and 7.38 ± 3.00, respectively, with higher scores among rural residents (P=0.003). Respondents with a technical secondary school degree and below had the highest practice scores (8.45 ± 2.63). Similar to COVID-19-related attitude scores, the higher the level of education, the lower the COVID-19-related practice score (*P* < 0.001). Respondents with monthly income 3,001–5,000 RMB and 5,001–10,000 RMB had mean practice scores 7.27 ± 2.98 and 7.18 ± 3.10, respectively (*P* = 0.003). Physical laborers had the highest mean COVID-19 practice score (7.34 ± 3.08) and students had the lowest mean score (6.47 ± 3.04). Sex, age, residential area, education, monthly income, and occupation were factors significantly associated with COVID-19 practice score.

As for total KAP score, the average score was 21.93 ± 4.18, with men scoring higher (22.55 ± 4.06) than women (*P* < 0.001). Older respondents had a higher mean total score, with the highest among those over 50 years old (22.59 ± 4.29). Respondents living in rural areas had higher mean total scores (22.28 ± 3.90) than urban residents (*P* = 0.027). Respondents with higher education levels had lower total KAP scores, with the lowest among respondents with a master's degree or above (21.21 ± 4.36, *P* < 0.001). Respondents with monthly income 3,001–5,000 and 5,001–10,000 RMB had similar mean scores, which were higher than scores among respondents with monthly income 3,000 RMB and below and 10,000 RMB and above (*P* = 0.002). Physical laborers had the highest mean total KAP score (22.23 ± 4.00) and students had the lowest (20.91 ± 3.82) (*P* = 0.002). Sex, age, residential area, education level, monthly income, and occupation were factors significantly associated with COVID-19 total KAP score (Table 5).

**Table 5:** Comparison of COVID-19 related KAP scores between different characteristics.

**Characteristics**		**Number of** **participants** **(%)**	**Knowledge**			**Attitude**			**Practice**			**Total KAP**		
			**$\bar{x}$ ±s**	***t*** **/F**	***P***	**$\bar{\text{x}}$ ±s**	**t/F**	***P***	**$\bar{\text{x}}$ ±s**	**t/F**	***P***	**$\bar{\text{x}}$ ±s**	**t/F**	***P***
Gender	Male	857(39.5)	8.88 ± 0.87			6.16 ± 1.61			7.50 ± 3.01			22.55 ± 4.06		
	Female	1,314(60.5)	8.97 ± 0.74	6.027	0.014	5.84 ± 1.54	22.46	<0.001	6.72 ± 3.19	32.483	<0.001	21.53 ± 4.21	31.462	<0.001
Age-group (years)	18–30	639(29.4)	8.89 ± 0.76			5.67 ± 1.44			6.60 ± 3.08			21.16 ± 4.00		
	31–40	698(32.2)	9.04 ± 0.69			6.00 ± 1.56			6.98 ± 3.18			22.03 ± 4.25		
	41–50	528(24.3)	8.99 ± 0.78			6.14 ± 1.61			7.24 ± 3.10			22.37 ± 4.10		
	≥51	306(14.1)	8.69 ± 1.00	15.619	<0.001	6.21 ± 1.72	12.624	<0.001	7.70 ± 3.13	9.576	<0.001	22.59 ± 4.29	12.115	<0.001
Region	Urban	1,632(75.2)	8.95 ± 0.79			5.95 ± 1.57			6.91 ± 3.18			21.82 ± 4.26		
	Rural	539(24.8)	8.88 ± 0.81	3.244	0.072	6.01 ± 1.59	0.598	0.44	7.38 ± 3.00	9.059	0.003	22.28 ± 3.90	4.892	0.027
Education	Technical secondary school and below	253(11.7)	8.52 ± 0.97			6.46 ± 1.79			8.45 ± 2.63			23.42 ± 3.77		
	Junior College/University	1,559(71.8)	8.98 ± 0.74			5.91 ± 1.55			6.97 ± 3.12			21.86 ± 4.14		
	Master degree or above	359(16.5)	9.03 ± 0.76	42.169	<0.001	5.88 ± 1.45	14.206	<0.001	6.30 ± 3.29	36.802	<0.001	21.21 ± 4.36	22.057	<0.001
Monthly income/	≤ 3,000	290(13.4)	8.80 ± 0.77			5.82 ± 1.55			6.88 ± 3.10			21.49 ± 3.99		
China Yuan	3,001–5,000	524(24.1)	8.92 ± 0.87			6.05 ± 1.67			7.27 ± 2.98			22.24 ± 4.06		
	5,001–10,000	840(38.7)	8.97 ± 0.75			6.03 ± 1.57			7.18 ± 3.10			22.18 ± 4.14		
	>10,000	517(23.8)	8.98 ± 0.78	4.026	0.007	5.86 ± 1.50	2.59	0.051	6.63 ± 3.36	4.628	0.003	21.47 ± 4.41	5.14	0.002
Occupation	Physical labor	290(13.4)	8.81 ± 0.87			6.08 ± 1.51			7.34 ± 3.08			22.23 ± 4.00		
	Other	524(24.1)	8.78 ± 0.82			5.94 ± 1.66			7.04 ± 3.30			21.76 ± 4.31		
	Students	840(38.7)	8.84 ± 0.76			5.60 ± 1.31			6.47 ± 3.04			20.91 ± 3.82		
	Mental labor	517(23.8)	9.06 ± 0.74	20.32	<0.001	6.00 ± 1.58	3.921	0.008	7.03 ± 3.09	2.797	<0.039	22.09 ± 4.20	4.91	0.002

### Binary logistic regression analysis of factors associated with COVID-19-related KAP scores

In Logistic Regression Analysis, Compared With Women, men Had Higher KAP Scores (Odds Ratio [OR]: 1.59; 95% Confidence Interval, [95% CI]: 1.31–1.92; *P* < 0.001). Older Respondents Also Had Higher Scores (OR: 2.00; 95% CI:1.43–2.80; *P* < 0.001). Respondents With Higher Education Levels Had Lower KAP Scores (OR: 0.45; 95% CI:0.29–0.68; *P* < 0.001). Binomial Logistic Regression Analysis Showed That sex, age, and Education Level Were Significantly Associated With Total KAP Toward COVID-19 (Table 6).

**Table 6:** Comparison of different COVID-19-related knowledge, attitude, and practice scores according to different participant characteristics.

**Characteristics**		**Score of** **knowledge (≥7)**	**OR** **(95% CI)**	**P**	**Score of** **attitude (≥7)**	**OR** **(95% CI)**	**P**	**Score of** **practice (≥7)**	**OR** **(95% CI)**	**P**	**Score of** **total KAP (≥21)**	**OR** **(95% CI)**	**P**
Gender	Female (Ref)	1,287(97.9%)			283(21.5%)	1		755(57.5%)	1		785(59.7%)	1	
	Male	824(96.1%)			256(29.9%)	1.51(1.23–1.84)	<0.001	602(70.2%)	1.77(1.46–2.14)	<0.001	599(69.9%)	1.59(1.31–1.92)	<0.001
Age-group (years)	18–30 (Ref)	624(97.7%)	1		103(16.1%)	1		358(56.0%)	1		349(54.6%)	1	
	31–40	690(98.9%)	2.30(0.96–5.50)	0.061	187(26.8%)	1.67(1.24–2.24)	0.001	432(61.9%)	1.27(0.99–1.62)	0.058	460(65.9%)	1.56(1.21–2.00)	0.001
	41–50	514(97.3%)	1.11(0.52–2.37)	0.786	150(28.4%)	1.71(1.26–2.34)	0.001	342(64.8%)	1.40(1.07–1.83)	0.014	349(66.1%)	1.53(1.17–2.02)	0.002
	≥51	283(92.5%)	0.44(0.22–0.90)	0.024	99(32.4%)	1.92(1.35–2.72)	<0.001	225(73.5%)	1.85(1.33–2.58)	<0.001	226(73.9%)	2.00(1.43–2.80)	<0.001
Education	Technical secondary school and below (Ref)	233(92.1%)	1		94(37.2%)	1		203(80.2%)	1		199(78.7%)	1	
	Junior College/University	1,523(97.7%)	2.91(1.60–5.29)	<0.001	364(23.3%)	0.54(0.39–0.73)	<0.001	953(61.1%)	0.49(0.35–0.69)	<0.001	977(62.7%)	0.52(0.37–0.74)	<0.001
	Master degree or above	355(98.9%)	5.07(1.64–15.69)	0.005	81(22.6%)	0.52(0.35–0.76)	0.001	201(56.0%)	0.43(0.29–0.65)	<0.001	208(57.9%)	0.45(0.29–0.68)	<0.001
Occupation	Physical labor (Ref)	281(94.9%)			73(24.7%)	1		198(66.9%)			199(67.2%)		
	Other	515(96.4%)			131(24.5%)	1.07(0.77–1.51)	0.681	330(61.8%)			327(61.2%)		
	Students	175(98.9%)			23(13.0%)	0.90(0.51–1.59)	0.723	93(52.5%)			92(52.0%)		
	Mental labor	1,140(97.9%)			312(26.8%)	1.47(1.07–2.02)	0.017	736(63.2%)			766(65.8%)		

## Discussion

Public health education is an effective measure to prepare the population for a catastrophic health emergency so that individuals can take preventive measures to reduce the likelihood of contracting a deadly disease (26). In our study, the average correct response rate for knowledge related to COVID-19 was close to 90%. This may be related to health education and dissemination of information to the Chinese public, consistent with the results of two previous studies in China (14, 27). Our study results indicated that 91.2% of respondents were aware that COVID-19 is an acute viral infection, which was slightly lower than the rate (97%) reported by Raza et al. (28). However, most respondents were unaware of the severity of COVID-19 and that there was still no specific treatment available at the time of the survey. However, 99.95% of respondents knew that wearing a mask was one way to prevent COVID-19 infection, which was consistent with the findings of Salman et al. (29). Most participants (99.72%) believed that keeping a physical distance from other people was a good way to avoid COVID-19 infection, which was similar to previous KAP studies conducted in China, the United Kingdom, South Korea, Indonesia, and the United Arab Emirates during infectious disease outbreaks (16, 26, 30, 31). Participants correctly identified the symptoms of COVID-19 with a high accuracy rate of over 85%, indicating a good understanding of this information. However, most participants mistakenly believed that eating garlic and taking antibiotics could prevent SARS-CoV-2 infection. These findings highlight that the responsible authorities should improve the dissemination of correct COVID-19-related information to improve knowledge and practices and help people prevent COVID-19 infection.

In logistic regression analysis, sex, age, and education level were significantly associated with the total KAP toward COVID-19. The relationship between sex, age, education, and KAP was consistent with attitudes and practices. The ORs indicated that male sex and age were predictors of high total KAP, and education level was negatively associated with high total KAP. Women scored higher than men for COVID-19-related knowledge, which was consistent with past studies (17, 26, 32). Highly educated respondents with a master's degree or above had better knowledge levels than respondents with lower education levels. A study conducted in China and Iran had a similar result (14, 17). More highly educated individuals may have more comprehensive knowledge of COVID-19 obtained from multiple sources. Mental laborers had the highest COVID-19 knowledge scores among all occupations. More highly educated people usually work in professions requiring more rigorous training and qualifications, which might explain the higher levels of COVID-19 knowledge in this group. Lower knowledge levels may be the result of relying on less credible information sources, which should be addressed in a timely manner. We propose that health ministries and government agencies arrange awareness and educational campaigns to promote COVID-19 prevention and control.

Univariate analysis showed that men had higher COVID-19 attitude and practice scores than women. Men were more willing to be vaccinated than women, a finding supported by other evidence reported in the literature (33, 34). This may be owing to concerns among women about the safety and effectiveness of COVID-19 vaccines, which is consistent with research conducted in China on COVID-19 vaccination willingness, where men were more likely to be vaccinated against COVID-19 (35). Older respondents had higher practice and attitude scores than younger ones, possibly because older people feel that they are at higher risk of contracting COVID-19. This group also has more limited sources of knowledge about COVID-19 and vaccines, mostly official media, and little exposure to false information and rumors. This is consistent with findings reported by Lazarus et al. (36) and Nguyen et al. (37). Respondents with lower education levels and physical laborers had higher COVID-19 practice and attitude scores than those with higher education levels and mental workers. This finding was consistent with those of a survey by Al-Marshoudi et al. (38) showing that people with low literacy levels were more willing to be vaccinated than those with post-secondary school or higher education levels, and a previous study of influenza vaccination reported similar results (39). This may be because individuals with higher education levels or professional occupations are concerned about the safety and effectiveness of COVID-19 vaccines. These groups may also have greater access to misinformation about COVID-19 and may be more influenced by rumors. A similar effect was recognized in a study showing that misinformation regarding COVID-19 directly affects health care workers (40). This indicates that even educated people can be affected by rumors. Reuben et al. (41) stated that unclear information and negative attitudes may lead to suffering and panic during an epidemic (40, 42). The Health Committee and Centers for Disease Control should disseminate correct information about COVID-19 in a timely and effective manner and find ways to address the fabrication and spread of rumors. COVID-19 practice scores among people living in rural areas were higher than those of their urban counterparts. People in rural areas have relatively low education levels, and most are engaged in physical labor. Most rural residents receive COVID-19-related information through official channels and are less affected by rumors. By knowing people's awareness of COVID-19 and their views on COVID-19 vaccine, and adopting epidemic prevention and control measures, we can spread correct knowledge and formulate appropriate prevention strategies, which play a crucial role in the management of COVID-19.

To control the COVID-19 pandemic, government agencies must launch effective public health campaigns. There is an urgent need to implement awareness-raising interventions at community level to educate the public regarding precautionary measures like wearing masks, correct hand hygiene, and the importance of social distancing. Mass media campaigns, talks held at educational institutions, and health promotion programs to provide health-related recommendations in rural and urban areas and eliminate misinformation and rumors are an important way forward.

This study had several limitations. We conducted online convenient sampling, which is inferior to random sampling; thus, the sample may not well represent the general public in China. We administered a cross-sectional survey and confounding factors could not be controlled to determine a causal relationship. Cohort studies are needed in the future to obtain additional information regarding KAP among the public and factors affecting KAP with respect to COVID-19. Online data collection will miss individuals who cannot access the Internet, such as older people and residents of remote areas. Because participants in our study were from some areas that were not severely affected by COVID-19, our study findings are not generalizable to residents living in other areas of China. Future studies should recruit a more representative and larger participant pool. Our questionnaire was conducted from September to October 2020; at that time, the epidemic situation was relatively stable, but the vaccination situation in China has changed since then. Nearly the entire Chinese population has now been vaccinated; therefore, our study findings cannot well represent the current situation. It is necessary to investigate KAP with regard to COVID-19 at different periods during the ongoing pandemic.

## Conclusion

The findings of this study suggest that the general public in China has high levels of KAP regarding COVID-19 under the present conditions of regular prevention and control of COVID-19. Male sex and older age were predictors of high total KAP whereas education level was negatively associated with high total KAP. To improve public awareness regarding prevention and control of COVID-19, official and public social media platforms that are popular among Chinese people should be used to disseminate accurate information regarding COVID-19 prevention and control.

## Data availability statement

The datasets generated and analyzed during this study are not publicly available due to the institute's data security and sharing policy, but are available from the corresponding author on reasonable request.

## Ethics statement

Ethical review and approval was not required for the study on human participants in accordance with the Local Legislation and Institutional requirements. Written informed consent for participation was not required for this study in accordance with the National Legislation and the Institutional requirements.

## Author contributions

Conceptualization: HL. Methodology, formal analysis, and investigation: HL and JY. Writing—original draft preparation: JY. Writing—review and editing: HL, JY, YL, QH, and CS. All authors contributed to the article and approved the submitted version.

## Funding

This research was funded by Key Program of Health Commission of Zhejiang Province/Science Foundation of National Health Commission, grant number is WKJ-ZJ-2221; the Key Research and Development Program of Zhejiang Province, grant number is 2021C03200.

## Conflict of interest

The authors declare that the research was conducted in the absence of any commercial or financial relationships that could be construed as a potential conflict of interest.

## Publisher's note

All claims expressed in this article are solely those of the authors and do not necessarily represent those of their affiliated organizations, or those of the publisher, the editors and the reviewers. Any product that may be evaluated in this article, or claim that may be made by its manufacturer, is not guaranteed or endorsed by the publisher.
